# Identifying Clinical Criteria for an Expanded Targeted Approach to Screening for Congenital Cytomegalovirus Infection—A Retrospective Study

**DOI:** 10.3390/ijns9030040

**Published:** 2023-07-24

**Authors:** Maya Heled Akiva, Hannah Hyde De Souza, Valerie Lamarre, Isabelle Boucoiran, Soren Gantt, Christian Renaud, Fatima Kakkar

**Affiliations:** 1 Department of Pediatrics, Montreal Children’s Hospital, McGill University, Montreal, QC H4A 3J1, Canada; mayahe@assuta.co.il; 2 Sainte-Justine Research Center, Université de Montréal, Montreal, QC H3T 1C5, Canada; 3 Department of Pediatrics, Université de Montréal, CHU Sainte-Justine, Montreal, QC H3T 1C5, Canada; 4 Department of Obstetrics and Gynecology, Faculty of Medicine, Université de Montréal, Montreal, QC H3T 1C5, Canada; 5 Department of Microbiology and Immunology, Université de Montréal, CHU Sainte-Justine, Montreal, QC H3T 1C5, Canada

**Keywords:** congenital cytomegalovirus (cCMV), sensorineural hearing loss (SNHL), targeted screening, universal newborn screening

## Abstract

Targeted screening for congenital CMV infection (cCMV), which entails CMV testing of infants who fail newborn hearing screening (NBHS), has become common practice. However, this strategy misses nearly all infected infants with normal hearing at birth who are nonetheless at high risk of subsequent hearing loss and would benefit from timely cCMV diagnosis. The objective of this study was to identify expanded criteria predictive of cCMV to increase the scope and utility of targeted newborn CMV screening. In this retrospective study, 465 newborns were tested for cCMV at a single tertiary care center with a targeted screening program between 2014 and 2018. Twenty-two infants were diagnosed with cCMV, representing 0.2% of the 12,189 births over this period and 4.7% of the infants tested. The highest prevalence of cCMV infection was among infants tested because of primary maternal CMV infection (8/42, 19%), followed by failed initial NBHS (10/88, 11.4%), maternal HIV infection (3/137, 2.2%), and clinical suspicion alone (5/232, 2.2%). The symptoms with the highest prevalence of infection among all infants tested included an enlarged liver and/or spleen (33.3%) (3/9), followed by petechiae (33.3%), microcephaly (9.4%), direct hyperbilirubinemia (7.7%), thrombocytopenia (6%), and growth impairment (4.3%). In addition to CMV screening of newborns who fail the NBHS, these data suggest that certain clinical signs of cCMV—in particular: thrombocytopenia, growth impairment, and HIV exposure in pregnancy—should be additional criteria for expanded targeted newborn CMV screening, where universal screening is not yet the standard of care.

## 1. Introduction

Cytomegalovirus (CMV) is the most common congenital infection, occurring in roughly 0.4% to 0.64% of live births in North America and Europe [1,2,3,4]. The majority of infants with congenital CMV infection (cCMV) are asymptomatic at birth; only between 10–15% are born with symptoms that may include intrauterine growth retardation, microcephaly, intracranial calcification, jaundice, hepatosplenomegaly, thrombocytopenia, and petechiae [5]. cCMV is a common cause of childhood neurodevelopmental disabilities, the most frequent of which is sensorineural hearing loss (SNHL) that may be present at birth or develop during the first several years of life [6]. Importantly, even children who have asymptomatic cCMV are at risk for developing late-onset SNHL [7].

The definitive diagnosis of cCMV requires the detection of the virus in a sample collected within the first 3 weeks of life to differentiate it from post-natal infection, which is not associated with the same neurologic sequelae [8]. In the absence of newborn CMV screening, the majority of infected children, including many who are symptomatic, are not diagnosed, likely because the clinical presentation is non-specific and often subtle [9,10]. This results in missed opportunities to provide beneficial directed care, including close audiologic follow-up, speech and language therapy, and hearing aids or cochlear implants for children who develop SNHL [11,12,13]. Universal newborn CMV screening programs would identify the greatest number of infected children and therefore provide the greatest benefit [11,13]. However, universal screening is not yet the standard of care in most countries worldwide. In North America, universal cCMV screening has only been implemented in the Canadian provinces of Ontario and Saskatchewan, and in the state of Minnesota (United States).

As an interim measure, many jurisdictions have implemented targeted screening programs, which test for CMV infection following a failed newborn hearing screen (NBHS). Although this approach requires a smaller investment and facilitates the identification of cCMV as the cause of SNHL when present at birth, targeted screening fails to identify large numbers of children, as the vast majority do not have SNHL at birth [14,15]. Importantly, a large study of universal newborn CMV screening found that targeted screening would have missed 43% of SNHL that developed during early infancy, the most critical period for speech and language development [16]. As a result, where universal newborn CMV screening is not available, additional criteria to expand the sensitivity of the targeted screening approach would be valuable. Since 2014 at our institution (CHU-Sainte-Justine, Montreal, QC H3T 1C5, Canada), a targeted program has been in place that includes maternal and infant indications in addition to a failed NBHS. The objective of this study was to identify which of the expanded criteria best supplemented targeted screening based on a failed NBHS alone.

## 2. Materials and Methods

This was a retrospective study of all newborns born between 1 March 2014 and 30 June 2018 at Sainte-Justine Hospital, a tertiary hospital and referral centre for high-risk pregnancies, that were tested for cCMV infection within the first 21 days of life. CMV testing was performed using PCR on either saliva, blood, or urine [17]. Patients were classified as having cCMV if they had a single positive PCR result on either urine or blood; a positive saliva PCR required a confirmatory urine or blood sample [8,18]. Medical charts of all newborns tested were reviewed by two independent reviewers to identify symptoms consistent with cCMV and the indications for testing, which included (1) failed NBHS (confirmed), (2) maternal HIV infection, (3) primary maternal CMV infection during pregnancy (suspected or confirmed), and (4) clinical suspicion based on symptoms at birth consistent with cCMV. Any discordant categorization was resolved by a third reviewer. Overlapping indications for testing were considered separately for the calculation of prevalence by testing indication.

Clinical symptoms consistent with cCMV were recorded for each case using the following definitions: microcephaly (head circumference < 3rd percentile), thrombocytopenia (platelet count <150 platelets/μL), direct hyperbilirubinemia (conjugated bilirubin >34 µmol/L (2 mg/dL)), hepatitis (ALT > 80 U/L), prematurity (gestational age at birth of <37 weeks). Growth impairment was defined as having one of the following: intrauterine growth retardation (IUGR; antenatal determination of estimated fetal weight or abdominal circumference <10th percentile for gestational age), SGA (birth weight < 10th percentile for GA), or low birth weight (<2500 g). The study was approved by the local Research Ethics Board. The most common criteria prompting testing, and the proportion of infected children with a given testing criterion, were described.

## 3. Results

From 1 March 2014 through 30 June 2018, a total of 12,189 infants were born at CHU-Sainte-Justine; of those, 465 (3.8%) were tested for CMV within the first 21 days of life. Twenty-two infants were diagnosed with cCMV (Table 1), representing 0.2% of all births and 4.7% of infants tested for CMV.

**Figure 1:**
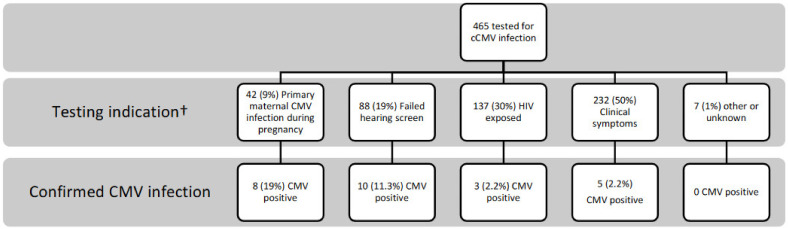
Confirmed newborns with cCMV infection according to testing indication. † Infants with multiple indications (n = 41) are represented in multiple categories. CMV—cytomegalovirus; cCMV—congenital CMV infection; HIV—human immunodeficiency virus.

The main indications for testing included suspected or confirmed primary maternal CMV infection during pregnancy (N = 42; 9%), failed initial NBHS (N = 88; 19%), maternal HIV infection (N = 137; 30%), and the presence of clinical symptoms other than a failed NBHS (N = 232; 50%). For 7 newborns, the clinical indication for testing could not be determined via chart review. For 41 infants, multiple indications for testing were identified, including combinations of failed hearing screen, suspected or confirmed primary maternal CMV infection during pregnancy, and/or intrauterine HIV exposure (See Figure 1 and Table 2).

The highest prevalence of cCMV was among infants tested because of primary maternal CMV infection (8/42; 19%), followed by failed hearing screen (10/88; 11.4%), maternal HIV infection (3/137; 2.2%), and newborn symptoms (5/232; 2.2%). Irrespective of indications for testing, 71.8% (334/465) of all infants tested had at least one clinical symptom compatible with cCMV, including the 69.3% (61/88) of newborns who failed their NBHS. The specific symptoms with highest proportions of infected infants, regardless the indication for testing, included enlarged liver and/or spleen (3/9; 33.3%), petechiae (4/12; 33.3%), microcephaly (3/32; 9.4%), direct hyperbilirubinemia (1/13; 7.7%), thrombocytopenia (8/132; 6%), growth impairment (11/256; 4.3%), hypotonia (3/74; 4%), and prematurity (3/106; 2.8%) (Table 2).

Among the 232 newborns tested due to newborn symptoms alone (no failed NBHS, suspicion of primary maternal CMV infection or maternal HIV infection), the highest proportions of cCMV were seen with an enlarged liver and/or spleen (14.3%), petechiae (12.5%), microcephaly (6.3%), thrombocytopenia (3.9%), growth impairment (2.9%), and prematurity (2.5%) (Table 3). The majority (159/232, 69%) had two or more symptoms compatible with cCMV and, of these, infection was confirmed in 4/159 (2.5%) (all 4 presented with growth impairment and thrombocytopenia.) Only 73 infants were tested because of an isolated symptom, of whom only 1 (1.3%) was confirmed to be infected (isolated growth impairment).

## 4. Discussion

In this study, we identified testing criteria and clinical symptoms commonly associated with cCMV infection in a cohort of newborns at a tertiary care maternal–child health center in Canada. Retrospective evaluation of a targeted testing program alongside symptoms-based testing demonstrated that expanded clinical indications (maternal HIV infection, suspected or confirmed primary maternal CMV infection during pregnancy, or symptoms compatible with cCMV infection) allowed better detection of cCMV than standard targeted testing programs. The highest proportion of cCMV was among infants tested due to a suspicion of primary maternal infection during pregnancy, followed by a failed hearing screen, HIV exposure, and the presence of two or more symptoms compatible with CMV. Strikingly, the traditional indication for targeted testing—a failed NBHS—identified only a single case of cCMV with true isolated SNHL with no other clinical symptoms consistent with cCMV infection.

Some clinical symptoms of cCMV such as hepatosplenomegaly and microcephaly are well-recognized although less frequent manifestations of cCMV infection and should always prompt CMV testing. Other clinical symptoms of cCMV infection, such as growth impairment (IUGR or SGA), thrombocytopenia, and direct hyperbilirubinemia, are less specific and more common, and their presence does not routinely result in CMV testing. Given that IUGR and thrombocytopenia were found in 2.9% and 3.9% of infants with confirmed cCMV tested due to symptoms, and 4.3% and 6% of infants tested due to all indications, these findings should trigger CMV testing. Direct hyperbilirubinemia was frequent among infants with cCMV (7.7%) and should also prompt CMV testing, though it was not found in isolation in this cohort.

Overall, in our study, 5 of the 22 (22.7%) cases were diagnosed solely based on clinical suspicion. However, the majority of positive cases (68.2%) had, in retrospect, at least one or more symptoms compatible with cCMV, suggesting that many clinical symptoms are not recognized as manifestation of cCMV due to their non-specific nature. In this respect, expanding targeted screening to include clinical symptoms potentially associated with cCMV (e.g., SGA) could potentially increase case detection.

On the other hand, 32.8% of the infants diagnosed had no clinical symptoms and would have been missed by symptoms-based testing. This reinforces the need for more widespread systematic screening criteria that do not depend on clinical suspicion alone. First, CMV testing of all HIV-exposed newborns, while a common practice in many Canadian institutions and recommended in the most recent Canadian guidelines [19], is not standard practice across countries and settings, specifically in areas of high HIV prevalence. Longitudinal birth cohorts have demonstrated a high risk of cCMV infection among children who were HIV-exposed though uninfected (CHEU), with rates increasing along with maternal CMV seroprevalence. While the overall prevalence of cCMV in the general population is estimated at 0.64% [1], the reported birth prevalence is 4–26% among children living with HIV and 2–7% among CHEU [20,21]. Low maternal CD4 cell counts and/or a high HIV viral load have been associated with an increased risk of cCMV. With the current recommendations of lifelong antiretroviral therapy for all people living with HIV [22], fewer women have a detectable viral load and HIV-associated immunosuppression during pregnancy. It is therefore assumed that the risk of cCMV in CHEU is significantly lower than previously estimated, such that cCMV screening for all CHEU infants is not routine. However, in this cohort of women with HIV, where all women had access to effective ART with 88% virally suppressed at time of delivery [23], 2.2% of exposed infants were diagnosed with cCMV. CHEU have now been shown across multiple cohorts both in resource-rich and resource-limited settings to be at an increased risk of speech and language delay and adverse neurodevelopmental outcomes [20]—given the potential contribution of cCMV infection, these data suggest that cCMV screening should be prioritized for newborns exposed to maternal HIV, where resources are available.

The highest risk of cCMV infection in our cohort was seen among cases with suspected maternal primary infection in pregnancy. Maternal screening for CMV infection is not routine across many jurisdictions, including our own, and as such, primary infection is not easily identified. The large number of suspected cases in our cohort is explained by the fact that our center is the largest referral center in the province for high-risk pregnancies, with a dedicated maternal infectious diseases clinic for suspicion of infections including CMV during pregnancy. While this criterion was the primary testing indication for <10% of cases, it resulted in 19.1% of newborns confirmed positive. This is unsurprising considering that primary infection with CMV during pregnancy poses a risk of intrauterine transmission of 30% to 40% [24]. Though there remains considerable debate on screening for maternal primary infection in pregnancy, with recent data that supports the use of valacyclovir for women with primary infection in the first trimester to reduce the risk of vertical transmission [25], it has been adopted by certain jurisdictions and proposed in the most recent Canadian guidelines [26]. In our study, suspicion of maternal primary infection during pregnancy prompted neonatal CMV testing in 8 of the 22 diagnosed newborns (Table 1), 4 of whom would have been missed by targeted testing (failed hearing or HIV exposure) and symptoms-based screening. Although this approach may be promising for selected low-CMV-seroprevalence populations, intrauterine transmission can also occur from non-primary infections. Prenatal screening of mothers for CMV would not be effective in these cases, and as such, additional post-natal screening programs for cCMV remain essential [15].

Finally, 11.3% infants of the 88 newborns who failed the initial NBHS in our cohort were diagnosed with cCMV. This is higher than has been previously reported (4.3–8.3%) by targeted hearing programs [14,27,28,29,30]. While the proportion of infants who failed screening (0.7%) is similar to previously reported results [0.8–1%] [16], the higher proportion of infants diagnosed with cCMV in our study may be in part explained by the fact that our center is the largest referral center in the province for high-risk pregnancies. This may result in a population of newborns at higher risk for cCMV infection and is therefore not reflective of the yield from targeted hearing screening in lower-risk settings.

We previously published on the use of viral load as a predictor of symptoms in newborns with cCMV [17], on a subset of cases (n = 11) also included in the current study who had viral load testing performed at time of diagnoses. Interestingly, in that study, which also included cases referred to our outpatient clinic but not born at our center (n = 47), the most commonly reported symptoms that prompted testing included CNS findings (52%), followed by a failed hearing screen (38.3%), IUGR (38.3%), and thrombocytopenia (36.3%), indicative of more moderately to severely symptomatic cCMV disease than reported in the current study. This is likely because targeting (routine or expanded) was not yet the standard of care in the referring centers; as such, the infants identified and referred for management were primarily due to the presence of symptoms. Similarly, in a separate sub-study from this same cohort comparing head ultrasound to magnetic resonance imaging, which included only infants with sequential neuroimaging (n = 37) [31], the majority of newborns were also diagnosed due to the presence of symptoms (58.7%). This is probably because symptomatic infants are more likely to have CNS involvement and require sequential neuroimaging. The current study evaluated only infants born at our center and excluded those referred to us, thereby reflecting more accurately a single-center targeted testing program.

Though there is currently little published data on expanded targeted testing programs, our results are very similar to a recently published study assessing a protocol for expanded targeted testing in the state of Utah. Using 10 criteria in addition to a failed newborn hearing screen as an indication for cCMV testing, the case detection rate increased to 37.5 per 100,000 live births compared to only 12.7 cases per 100,000 live births with only targeted hearing screening [32].

The primary limitation of this study is its single-center retrospective nature. The indications for CMV testing were based on patient chart review and relied on the accuracy of patient files to document physician reasoning. Thus, the reason for CMV testing may not have been comprehensively captured. In our cohort, the overall prevalence of diagnosed cCMV was 0.2%, which is much lower than the previously reported prevalence of 0.4% to 0.64% [1,2,3,4]. While the true incidence of cCMV in the province Quebec is unknown, studies in our province have shown low maternal CMV seroprevalence of 23% to 40% [33,34], which is associated with lower rates of cCMV [4] and may account for the low prevalence observed in our cohort. Accordingly, we assume that most infants with cCMV were not tested, and that those captured in this study are an underestimate of the true numbers at our center. Finally, as a retrospective, descriptive study that looked only at tested newborns, some infants with the described symptoms (specifically IUGR or thrombocytopenia) but who were not tested for CMV would not have been captured; therefore, the true frequency according to clinical symptoms is not known. Finally, as a single-center study based out of a tertiary care referral center for perinatal infectious diseases, our results may not be reflective of other birth hospitals given the potential referral bias from high-risk cases.

## 5. Conclusions

These findings suggest that expanding the indications for targeted screening (to include, in addition to failed NBHS: HIV exposure, primary maternal CMV infection during pregnancy, and high-yield neonatal symptoms consistent with cCMV) could increase the likelihood of identifying infected infants compared to current targeted screening programs. Pending universal screening of all newborns, systematically increasing the indications for targeted screening may improve the early diagnosis of cCMV. Further work on the feasibility, implementation, and cost-effectiveness of such programs across different nursery settings is necessary to help guide clinical practice.

## Figures and Tables

**Table 1:** Characteristics of the 22 newborns diagnosed with cCMV.

CMV Positive Infants	Reason for Testing	Newborn Hearing Screen Results	Symptoms
1	Primary maternal CMV infection	Pass	-
2	Primary maternal CMV infection	Bilateral fail	Growth impairment, Thrombocytopenia, Petechia
3	Primary maternal CMV infection	Pass	-
4	Primary maternal CMV infection	Unilateral	Hepatomegaly, Hypotonia
5	Primary maternal CMV infection	Pass	Thrombocytopenia
6	Primary maternal CMV infection	Pass	-
7	Primary maternal CMV infection	Pass	-
8	Primary maternal CMV infection	Unilateral fail	Growth impairment, Microcephaly, Petechia
9	Intrauterine HIV exposure	Pass	Growth impairment, Prematurity
10	Intrauterine HIV exposure	Unilateral fail	-
11	Intrauterine HIV exposure	Pass	-
12	Clinical symptoms	Pass	Growth impairment
13	Clinical symptoms	Pass	Growth impairment, Thrombocytopenia, Prematurity
14	Clinical symptoms	Pass	Growth impairment, Thrombocytopenia, Microcephaly
15	Clinical symptoms	Pass	Growth impairment, Thrombocytopenia, Prematurity, Hypotonia
16	Clinical symptoms	Pass	Growth impairment, Thrombocytopenia, Petechia, Splenomegaly
17	Failed hearing screen	Unilateral fail	Pneumonitis, Thrombocytopenia
18	Failed hearing screen	Unilateral fail	Growth impairment, Microcephaly
19	Failed hearing screen	Unilateral fail	-
20	Failed hearing screen	Bilateral fail	Growth impairment, Thrombocytopenia, Petechia, Hyperbilirubinemia, Splenomegaly
21	Failed hearing screen	Bilateral fail	Growth impairment
22	Failed hearing screen	Unilateral fail	Hypotonia

CMV—cytomegalovirus; HIV—Human immunodeficiency virus. Growth impairment—includes newborns with intrauterine growth retardation (IUGR), small for gestational age (SGA), or low birth weight (LBW).

**Table 2:** CMV infection according to indication of testing.

Indications for Testing	Tested (N)	Positive (N)	Percentage (%)
Maternal primary CMV infection (total)	42	8	19.1
Maternal primary CMV infection only	29	5	17.2
Maternal primary CMV infection + failed hearing screen	8	3	37.5
Maternal primary CMV infection + HIV exposed	5	0	0
Failed hearing screen (total)	88	10	11.4
Failed hearing screen only	52	6	11.5
Failed hearing screen + Maternal primary CMV infection.	8	3	37.5
Failed hearing screen + HIV exposed	28	1	3.6
HIV exposed (Total)	137	3	2.2
HIV exposed only	104	2	1.9
HIV exposed + Failed hearing screen	28	1	3.6
HIV exposed + Maternal primary CMV infection	5	0	0
Clinical Symptoms †	232	5	2.2

† Indication for testing was reported as clinical symptoms only if no other indication was present. cCMV—congenital cytomegalovirus; CMV—cytomegalovirus; HIV—human immunodeficiency virus.

**Table 3:** Clinical symptoms with highest yield for cCMV infection.

Indication for Testing	Symptoms Alone (n = 232)			All Indication (n = 465)		
Symptom	Newborns Tested	CMV+		Newborns Tested	CMV+	
	N	N	%	N	N	%
Enlarged liver and/or spleen	7	1	14.3	9	3	33.3
Microcephaly	16	1	6.3	32	3	9.4
Growth impairment	172	5	2.9	256	11	4.3
Thrombocytopenia	102	4	3.9	132	8	6
Petechia	8	1	12.5	12	4	33.3
Hypotonia	47	1	2.1	74	3	4
Prematurity	81	2	2.5	106	3	2.8
Direct hyperbilirubinemia	10	0	0	13	1	7.7

Growth impairment—includes newborns with intrauterine growth retardation (IUGR), small for gestational age (SGA), or low birth weight (LBW).

## Data Availability

The data presented in this study are available on request from the corresponding author.
